# Do Concurrent Peri-Implantitis and Periodontitis Share Their Microbiotas? A Pilot Study

**DOI:** 10.3390/dj12040113

**Published:** 2024-04-18

**Authors:** Ana Parga, José Manuel Pose-Rodríguez, Andrea Muras, María Baus-Domínguez, Paz Otero-Casal, Marcos Luis Ortega-Quintana, Daniel Torres-Lagares, Ana Otero

**Affiliations:** 1 Department of Microbiology and Parasitology, CIBUS-Faculty of Biology, University of Santiago de Compostela, 15782 Santiago de Compostela, Spain; ana.parga.martinez@usc.es (A.P.); andrea.muras@unavarra.es (A.M.); 2 Aquatic One Health Research Center (iARCUS), Edificio CIBUS, Campus Vida, Universidade de Santiago de Compostela, 15782 Santiago de Compostela, Spain; 3 Department of Surgery and Medical-Surgical Specialities, Faculty of Medicine and Odontology, University of Santiago de Compostela, 15782 Santiago de Compostela, Spain; josemanuel.pose@usc.es (J.M.P.-R.); mortegaquintana@gmail.com (M.L.O.-Q.); 4 Department of Stomatology, Faculty of Odontology, University of Seville, 41009 Sevilla, Spain; mbaus@us.es (M.B.-D.); danieltl@us.es (D.T.-L.); 5 Unit of Oral Health, Santa Comba-Negreira, (CS) SERGAS, 15840 Santiago de Compostela, Spain

**Keywords:** peri-implantitis, periodontal disease, oral microbiome, Illumina, microbiology, collection method

## Abstract

The microbial compositions from concurrent peri-implant and periodontal lesions were compared, since the results reported in the literature on the etiological relationship between these oral pathologies are contradictory. Microbial compositions from nine patients were evaluated using Illumina MiSeq sequencing of 16S rRNA gene amplicons and Principal Components Analysis. Comparisons between the use of curettes or paper points as collection methods and between bacterial composition in both pathologies were performed. Paper points allowed the recovery of a higher number of bacterial genera. A higher bacterial diversity was found in peri-implantitis compared to periodontal samples from the same patient, while a greater number of operational taxonomic units (OTUs) were present in the corresponding periodontal samples. A higher abundance of oral pathogens, such as *Porphyromonas* or *Treponema*, was found in peri-implantitis sites. The opposite trend was observed for *Aggregatibacter* abundance, which was higher in periodontal than in peri-implantitis lesions, suggesting that both oral pathologies could be considered different but related diseases. Although the analysis of a higher number of samples would be needed, the differences regarding the microbial composition provide a basis for further understating the pathogenesis of peri-implant infections.

## 1. Introduction

In recent years, peri-implant infectious diseases (PIID), including peri-implant mucositis (PIM) and peri-implantitis (PI), have emerged as significant new oral health problems. While PIM, which affects up to 30% of implants [1], is described as a localised inflammation of the mucosa surrounding the implant, PI, in addition, presents a progressive loss of implant-supporting bone that can lead to implant loss [2,3,4]. Therefore, although they are defined as chronic inflammatory disorders of bacterial aetiology, a better understanding of their pathogenesis will be key not only for treatment but also in their prevention.

Previously, PIID were thought to be closely related to periodontal diseases (PD), as both pathologies are triggered by dysbiotic biofilms and present microbial and clinical similarities [5,6]. In this sense, PIM and PI were considered analogues of gingivitis and periodontitis, respectively. Patients with a periodontal history appear to be more susceptible to developing PI than healthy individuals [3,7]. In addition, different periodontal pathogens such as *Prevotella*, *Porphyromonas*, *Fusobacterium*, or *Veillonella* are also present in PI, although their role in this pathology is still unclear, as they could be identified in oral niches other than PD and PI [6].

However, both types of lesions present important histological differences. Marginal bone loss with crater-like defects is distinctive for peri-implantitis, which, together with the inflammatory infiltrate extending apically to the pocket epithelium and a large number of plasma cells and osteoclasts referring to active bone destruction, are substantially different features from what is observed around teeth with periodontitis [8]. This is also reported in the case of mucositis, with soft tissue biopsies taken from around implants showing a larger size of inflammatory infiltrate with a greater number and density of polymorphonuclear cells extending apically to the pocket epithelium [8]. In addition, previous work using metatranscriptomic analysis indicated that PD and PI lesions showed different wound healing, cell adhesion, complement activation, and other immune responses [9].

Evaluation of the results reported in previous studies on PI- and PD-associated microbiota is complicated by the heterogeneity of analytical methods, donor selection, and the use of different sequencing techniques and collection methods. These differences in methodology could be responsible for the contradictory conclusions reached by different authors. So far, no consensus has been reached on which pathogens are actually related to PI, and contradictory information on PI- and PD-associated pathogens has been found in the literature over the last decade.

Several studies have used culture-based methods, DNA–DNA hybridisation techniques, or 16S rRNA gene amplicon sequencing to increase knowledge about the pathogens associated with PIM infections [10,11,12,13]. Research using real-time PCR has associated the typical periodontopathogens *Porphyromonas gingivalis* and *Fusobacterium nucleatum* with PD but not with PI [14]. However, the information obtained with these closed methods is limited, as they target specific, mostly culturable, bacterial taxa.

The use of next-generation sequencing (NGS) techniques to investigate the 16S rRNA gene has been instrumental in eliminating the biases of closed approaches, allowing the description of greater microbial diversity at PI sites compared to PD sites from the same patient [12,15]. Some studies support that oral biofilm is more diverse in PI lesions than in PD sites [15,16,17]. The use of 16S rRNA gene clone libraries showed higher microbial diversity in samples from the same patient when comparing implants with peri-implantitis and healthy implants [12] and a higher abundance of the phylum Bacteroidota as well as members of the red complex [18]. Pyrosequencing analysis has also indicated that the microbiota associated with PI and PD are sufficiently different to be considered two distinct diseases using samples from the same donor [19] or from different patients [20,21]. In contrast, other studies reported that the composition of the microbiota of PI and PD samples using 16S rRNA gene pyrosequencing is reasonably similar in the same subject [22,23]. On the other hand, a comparison of healthy implants with PIM and PI sites from different individuals using 16S rRNA gene pyrosequencing found a higher diversity in defective dental devices than in healthy implants and suggested the possible role of *Eubacterium* in the development of PI [21]. Other studies also support the idea that oral biofilm is more diverse in PI lesions than in PD sites [15,16,17], although, in a previous study, the opposite was observed in nine partially edentulous patients diagnosed with gingivitis and mucositis [24].

According to the results described by open 16S rRNA amplicon sequencing studies, a group of “core” taxa can be found in both PD and PI, comprising *Fusobacterium* spp., *P. gingivalis*, *Eubacterium* spp., *Parvimonas micra*, *Neisseria subflava*, *Streptococcus* spp., and *Rothia* spp., while different sets of bacterial taxa are often differentially associated with these conditions with varying degrees of statistical significance [25,26]. It is assumed that the heterogeneity found in NGS studies is due to differences in patient recruitment criteria, clinical parameters, interpersonal variation, the sampling technique used, and, evidently, the sequencing technology selected [27,28].

The main objective of the present study was to investigate and compare the microbial composition present in lesions of periodontal disease and peri-implantitis using next generation sequencing (NGS) technology. To achieve this objective, we specifically set out to identify the bacterial species present in concurrent PD and PI lesions of nine subjects in order to characterise the microbial diversity that would allow us to know the predominant species in each type of lesion and compare their relative distribution. For the analysis of the diversity and relative abundance of the microbial communities, a quantitative analysis of the microbial species, including richness, fairness, and community structure, was performed using paper tips to sample the subgingival biofilms. Overall, this study aims to provide a more complete understanding of the microbial composition of PD and PI lesions, which could contribute to the development of more accurate and personalised approaches to the diagnosis and treatment of these chronic oral diseases.

## 2. Materials and Methods

### 2.1. Type of Study

This is a prospective observational, cross-sectional pilot study approved by the Ethical Committee of Clinical Investigations of Galicia (protocol 2018/560) and complies with all the guidelines of the Helsinki Declaration: Ethical Principles for Ethical Research Involving Human Subjects [29], where the only procedure performed on patients was the collection of subgingival biofilm samples from patients with concurrent periodontal and peri-implant diseases for their subsequent analysis. Samples and clinical data of donors were collected in a fully anonymised manner, and the extracted DNA samples were destroyed after sequencing.

All patients signed an informed consent form based on this study and understood and accepted the type of treatment performed on them.

### 2.2. Study Participants

Patient recruitment was carried out at the Dental Clinic of the Faculty of Medicine and Dentistry of the University of Santiago de Compostela.

The description of the pathologies, the analysis of the clinical history, and the selection of the cases of peri-implant diseases was carried out by the team of odontologists participating in this study (J.M.P.-R. and P.O.-C.). Selected patients were diagnosed with PI per the guidelines described by Tonetti and Sanz (2019) [30].

The inclusion criteria were as follows: (1) patients over 18 years of age, (2) patients with simultaneous periodontal disease and peri-implant disease with bone loss greater than 3 mm, (3) patients who had received their implant at least one year before and whose PI was present at least 1 month ago, (4) patients in good general health, and (5) patients who did not present diseases requiring therapies that interfere with the pathogenesis or resolution of the pathology.

The exclusion criteria were: (1) pregnant patients, (2) patients with diabetes mellitus, and (3) patients taking medication related to gingival or bone metabolism disorders. Patients receiving antibiotic treatment were not excluded from this pilot study in order to assess if this treatment could cause evident convergent or divergent effects on the microbiota.

Sampling is limited to subgingival dental plaque and does not involve the patients’ gingival tissues.

### 2.3. Sampling and Microbial DNA Extraction

Mechanical debridement was applied to PD and PI lesions before sampling. For patients 1 and 2, subgingival biofilm samples were collected using two different methods: paper points and curette. For these two patients, two paper points were first inserted into each sampled site for 20 s in the deepest portion of the PI and PD pockets. Sterile Teflon Implant Deplaquer curettes (equivalent to Gracey 7/8) were gently inserted into the PD and PI pockets twice. For the remaining seven patients, only paper points were used for sampling. Curette-obtained samples and paper points were introduced in Eppendorf tubes containing 350 µL of the MBL solution of the “DNeasy PowerBiofilm Kit” (Qiagen^®^, Hilden, Germany) and kept at −20 °C until extraction.

### 2.4. Microbial DNA Extraction

A genomic DNA (gDNA) extraction was performed using the “DNeasy PowerBiofilm Kit” (Qiagen^®^) following the manufacturer’s instructions (https://www.qiagen.com, accessed on 7 April 2024). This kit has previously provided good recovery rates in oral biofilms, even in those dominated by Gram-positive species [31] and in supragingival biofilm samples with adequate recovery of Gram-positive members [32]. Briefly, samples were mechanically analysed before removing protein and inhibitor contaminants. Large insoluble molecules were eliminated using pH-driven precipitation, and the total gDNA was retained on a silica filter column by centrifugation. Eluted gDNA concentration was measured using a NanoDrop (ThermoScientific, Waltham, MA, USA).

### 2.5. Library Preparation

The library preparation, sequencing, and bioinformatics analysis were performed in the Foundation for the Promotion of Health and Biomedical Research of Valencia Region (FISABIO, ES, https://fisabio.san.gva.es/es/, accessed on 7 April 2024). Genomic DNA (5 ng/µL in 10 mM pH 8.5) was used to amplify the V3–V4 hypervariable regions of the 16S rRNA gene [33,34]. The primer sequences used in this protocol were 16S Amplicon 341F (TCGTCGGCAGCGTCAGATGTGTATAAGAGACAGCCTACGGG-NGGCWGCAG) and 805R (GTCTCGTGGGCTCGGAGATGTGTATAAGAGACAG-GACTACHVGGGTATCTAATCC). The libraries were prepared according to Illumina’s protocol (Part #15044223 Rev. A) and sequenced using a 2 × 300 base pair paired-end run on a MiSeq.

### 2.6. Bioinformatics and Microbial Diversity Analysis

The paired-end fastq files were processed as described elsewhere [31,32] using the prinseq-lite program [35] and the DADA2 pipeline [36]. Trimming and filtering of low-quality bases was performed. The amplicons were mapped for taxonomic affiliation using qiime2 and the database SILVA_release_132 [37]. All computations and statistics have been carried out with a pipeline written in RStatistics environment [38] using knitr, knitcitations, markdown [39,40,41] biostrings, and vegan [42]. The PCA was performed in RStatistics, and graphs were generated using ggfortify and ggplot2 [43,44].

## 3. Results

### 3.1. Study Participants

Nine patients (six females and three males) were recruited from the University Dental Clinic of the Faculty of Medicine and Dentistry of the University of Santiago de Compostela (Table 1).

Three patients were being treated with chlorhexidine (patient 8), antibiotics (patient 7), or both (patient 5) in the one month period prior to the sampling procedure.

### 3.2. Comparison of the Subgingival Microbial Diversity in Biofilms Recovered Using Paper Points or Curette Collection

Subgingival biofilms from PD and PI sites were sampled from two subjects (patients 1 and 2) using two different collection methods: paper points inserted into the deepest part of the pocket and collection with a curette. The microbial composition of the subgingival biofilms was examined by extracting the gDNA and sequencing the V3–V4 regions of the 16S rRNA gene to perform taxonomic assignments. Diversity was calculated as Shannon index values at the family, genus, and operational taxonomic unit (OTU) level.

**Figure 1:**
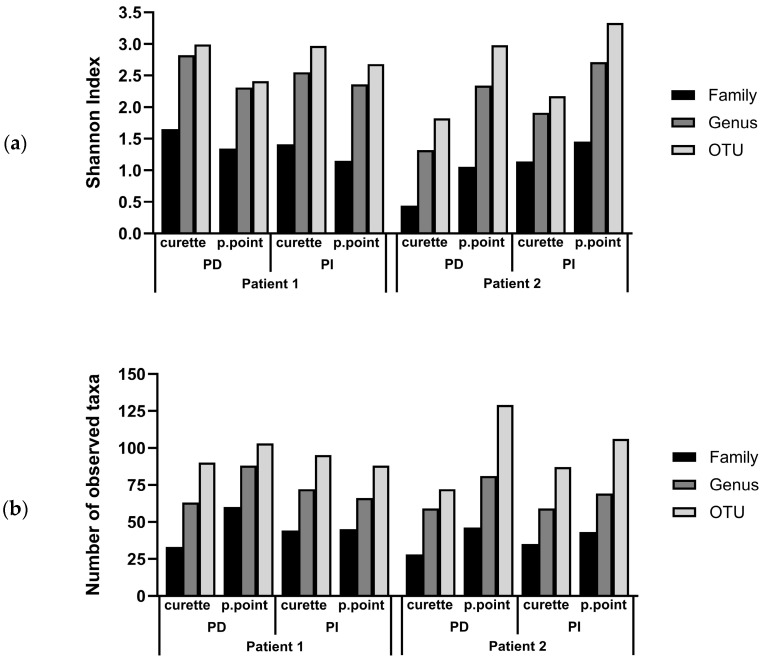
Comparison of the bacterial diversity of subgingival biofilms of two patients sampled with paper points (p.point) and a curette. Both periodontitis (PD) and peri-implantitis (PI) sites were sampled: (**a**) histograms display Shannon index values for each sample and collection method, at the family, genus, and operational taxonomic unit (OTU) level; (**b**) histograms display number of observed taxa at the family, genus, and OTU level.

Figure 1a displays the community diversity of each subgingival biofilm, comparing both sampling methodologies used, as well as comparing PD and PI sites for two patients. For patient 1, the PD subgingival biofilms retrieved with paper points displayed lower Shannon index values at the OTU level (2.41) than those retrieved with a curette (2.99). Conversely, for patient 2, the subgingival biofilms sampled using paper points displayed higher diversity values (2.98) than biofilms sampled with a curette (1.82). The same trend was observed in PI sites, with biofilms sampled from patient 1 using paper points displaying lower (2.68) diversity values than those sampled with a curette (2.97). The subgingival biofilms from patient 2 displayed higher diversity values when sampling was performed with paper points (3.33) compared to curette sampling (2.17).

When assessing the total number of taxa identified in the subgingival biofilms, collection of the biofilm with paper points consistently resulted in samples with a higher number of taxa retrieved, as compared to curette sampling, in PD sites of patient 1, and both PD and PI sites in patient 2 (Figure 1b).

**Figure 2:**
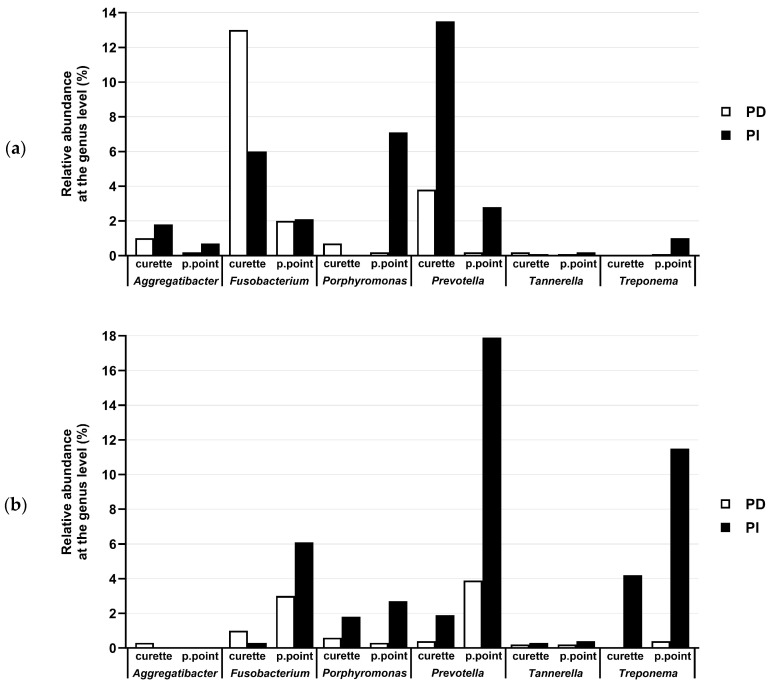
Relative abundances of the genera *Aggregatibacter*, *Fusobacterium*, *Porphyromonas*, *Prevotella*, *Tannerella*, and *Treponema* in periodontitis (PD) (white bars) and peri-implantitis (PI) (black bars) sites obtained from patient 1 (**a**) and patient 2 (**b**).

**Figure 3:**
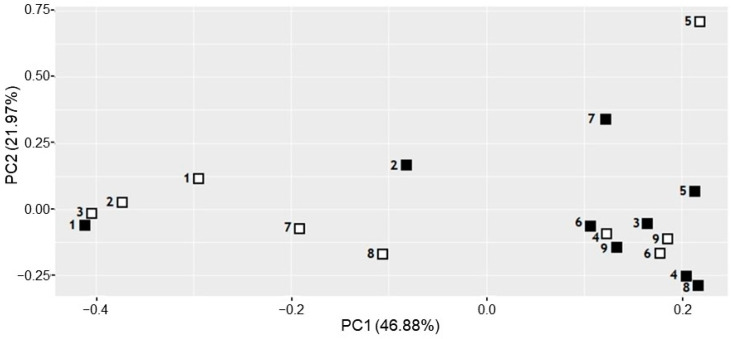
Principal component analysis (PCA) of the microbial composition of subgingival samples obtained from concurrent periodontitis (PD, white squares) and peri-implantitis (PI, black squares) lesions from nine subjects using paper points. The first principal component (PC1) is plotted on the *x*-axis and explains 46.88% of the observed variation. The second principal component (PC2) is plotted on the y-axis and explains 21.97% of the observed variation.

Paper points also allowed a higher proportion of periodontal pathogens to be retrieved in patient 2, while the opposite was observed in patient 1, in which sampling with a curette allowed higher proportions of the genera *Fusobacterium* and *Prevotella* (Figure 2). It is worth noting that, in subsequent examinations of the microbial composition of these samples, those from patient 1 presented a diverging trend compared to other patients (Figure 3).

### 3.3. Comparison of the Microbiota of PD and PI Sites Using NGS

Since the use of paper points allowed retrieving a higher number of OTUs and genera than the curette in almost all cases, this collection method was selected for further investigation of the microbial composition of PD- and PI-associated subgingival biofilms from seven additional subjects. The microbial composition of the samples was determined, as outlined above, by sequencing the V3–V4 regions of the 16S rRNA gene.

**Figure 4:**
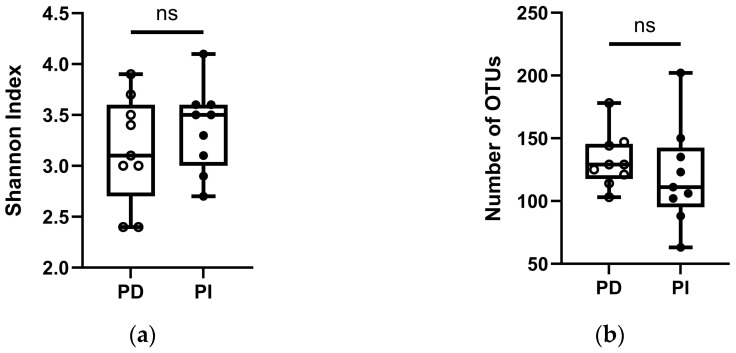
Comparison of bacterial diversity expressed using the Shannon index: (**a**) and number of taxa at the OTU level; (**b**) obtained in the samples from concurrent periodontitis (PD) and peri-implantitis (PI) sites in nine subjects. Samples were taken using paper points. Boxplots show minimum to maximum values of the Shannon index, and all samples are plotted as dots. Pairwise comparisons were performed using *t*-tests (*p* = 0.05). ns: not significant.

The analysis of the microbial diversity of the samples showed that for five subjects, the PI samples presented higher Shannon index values than the PD samples (Figure 4a). Only patients 6 and 7 had a higher Shannon index in the periodontal pocket (3.39 and 3.37) compared to their concurrent PI site (3.53 and 2.88). Patient 5, who had been prescribed antibiotics the week prior to sampling, presented the largest difference in Shannon index between the PD and PI samples. Despite obtaining a higher Shannon index in PI sites in most patients, a higher number of OTUs was found in PD sites in six out of nine patients (Figure 4b), with patients 4 and 9 showing a higher number of OTUs in PI sites, constituting the samples with the highest number of OTUs.

The bacterial community structure was further studied using a Principal Component Analysis (PCA) (Figure 3). The PCA demonstrated the influence of the sample origin on the microbial composition. More similarity was observed among the PI samples that clustered on the right side of the *x*-axis of the PCA, displaying the first principal component (PC1). Conversely, PD samples were found scattered throughout the *x*-axis. Concurrent PD and PI samples appear to be different for most subjects, discriminated mostly by PC1, although both PD and PI samples from patients 4, 6, and 9 clustered closely, indicating similar microbial composition (Figure 3). This clustering of PD and PI samples from the same subject was also observed in patient 1, whose samples were previously described as scarcely informative to distinguish the profiles of PD and PI samples obtained using different sampling techniques (Figure 1 and Figure 2). No divergent behaviour was observed in samples from patients that had been administered antibiotic or chlorhexidine treatment during the month before the sampling (Patients 7 and 8), while in patient 5, receiving both antibiotic and chlorhexidine treatments, PD and PI samples are more divergent on the PC2 axes (Figure 3).

**Figure 5:**
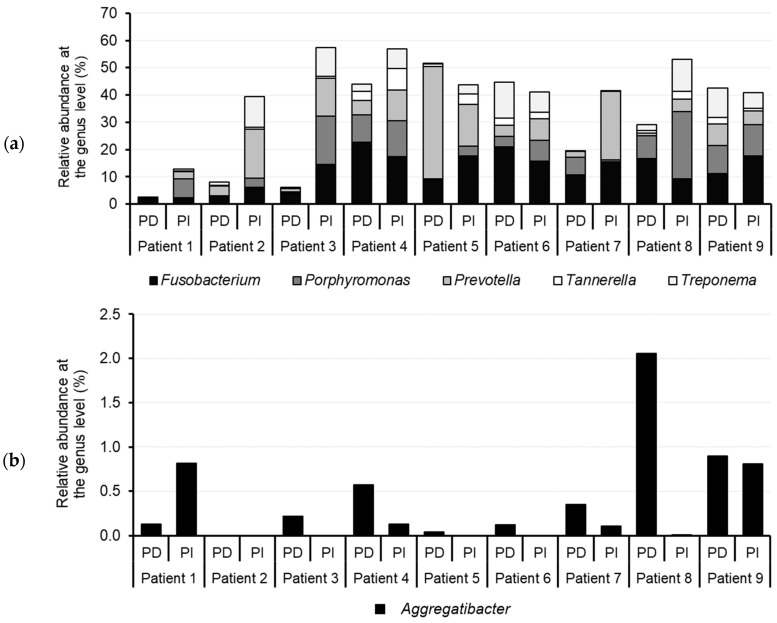
(**a**) Relative abundance, expressed in percentage, of the genera *Fusobacterium, Porphyromonas*, *Prevotella*, *Treponema*, and *Tannerella* in the samples from periodontitis (PD) and peri-implantitis (PI) sites obtained from nine different donors. (**b**) Relative abundance, expressed in percentage, of the genus *Aggregatibacter*.

Regarding the presence of pathogens identified in the samples, a high prevalence of important oral pathogens, including the genera *Fusobacterium*, *Porphyromonas*, *Prevotella*, *Treponema*, and *Tannerella* (Figure 5a), was observed in both PD- and PI-associated sites. Nevertheless, the abundance of these periodontopathogens was clearly higher in the PI sample compared to the PD sample from the same patient, except for patients 5, 6, and 9. Representatives of the genera *Porphyromonas*, *Tannerella*, *Prevotella*, and *Treponema* were more abundant in the PI sites compared to the PD sites. OTUs belonging to these important genera comprising oral pathogenic species represented at least 50% of the microbiota of the PI sites sampled from patients 3, 4, and 8. Additionally, *Fusobacterium* was also slightly higher in PI than in PD sites, except for patient 1. The opposite trend was observed for the genus *Aggregatibacter* (Figure 5b), whose abundance was higher in PD than in PI sites, although present at low relative abundance (0.008–2.06%) and not being identified in all samples.

## 4. Discussion

The aim of this study was to determine the degree of similarity of the microbiota associated with concurrent PI and PD infections. Given the discrepancies observed in previous studies regarding the most appropriate sampling model for subgingival sampling [45,46,47,48], the present study opted to analyse the microbiota using two methods of collecting samples from the same PI and PD lesions: curettes, and paper points. Previously published studies evaluated both methodologies to compare the microbial composition of gingival fluid and healthy tooth/implant surface [10], aggressive and chronic periodontitis samples [48], and, more recently, in gingivitis and periodontitis samples [49]. These studies demonstrated that the microbial profiles obtained using curettes differ from those collected with paper points [48,49], to which our results also subscribe.

The use of curettes as a collection method was suggested as more suitable for the assessment of periodontal microbiota using quantitative PCR due to less contamination with patient DNA [48]. However, in gingivitis and periodontitis samples from patients with rheumatoid arthritis, curettage was reported to yield higher bacterial diversity [49]. In contrast, paper points allowed the detection of several periodontopathogens, such as *A. actinomycetemcomitans*, *Tannerella forsythia*, and *Treponema denticola* [46,49].

In this study, sterile paper points were selected to analyse PD and PI samples, as this sampling technique allowed the highest number of OTUs in almost all samples compared to curettes. Furthermore, it has been described that paper point sampling provides a good representation of the outer biofilm layer and the free-floating bacteria present in the subgingival pocket [50]. Similarly, it should be noted that sample collection is easier and probably more reproducible with paper points.

Since hybridisation and PCR techniques can only detect preselected species, e.g., bacteria associated with periodontal disease, such as *P. intermedia*, *A. actinomycetemcomitans*, *P. gingivalis*, or *F. nucleatum*, the Illumina MiSeq sequencing technology was used to compare the microbial composition in the PI and PD samples in this study. This was because 20–60% of the oral microbiota is not culturable by standard laboratory methods [51]. This technique also enables a fast, effective, and comprehensive identification of the microorganisms present in the sample without requiring the prior targeting of a particular species. Because interpersonal variation is a potential confounding factor, only concurrent PD and PI lesions have been sampled here.

A comparison of the microbial composition of PI and PD samples from nine patients indicates that the associated bacterial communities are different, with the microbiota from PI sites being more similar to each other than those from the PD samples. This result is in line with a recent systematic review [51], which defines peri-implantitis as a distinct pathological entity from periodontitis, and presents a greater microbial heterogeneity. In contrast, another study using MiSeq sequencing showed that PD and PI sites within the same subject were reasonably similar in terms of bacterial diversity, suggesting that they should be considered the same pathology [22].

The fact that some authors conclude that the microbiome of peri-implant diseases is equivalent to that of periodontal diseases [23,51,52] may be due to the use of traditional microbiological methods. More recent study results report statistically significant differences between taxa from sites diagnosed with peri-implantitis versus periodontitis, affirming that the peri-implant microbiota is a microbiologically distinct ecosystem from periodontal microbiota [23]. A study sequencing 16S rRNA gene amplicons described differences in the “core” microbiota, defining each lesion while describing equivalent bacterial diversity values between PD and PI samples [19]. However, in the study by Yu et al., 2019 [23], where they compared the subgingival and submucosal microbiota of clinically defined diseased and healthy periodontal teeth and implants through Illumina MiSeq sequencing, they reported considerably different bacterial compositions between individuals, but with a relatively similar submucosal and subgingival microbiotas, with a core set of microorganisms shared by all patients studied, including *Streptococcus*, *Fusobacterium*, and *Veillonella*, among other species. However, a very recent systematic review by Kensara et al., 2024 [11], reports studies where periodontal pathogens were analysed and concludes that *P. gingivalis* is more frequent in periodontitis, while *Fusobacterium* is more frequent in peri-implantitis. These results are in line with other bacterial species from other included studies, and in contrast to other authors who reported that there were no significant gender differences between the two entities in the same patient. Regarding bacterial species, in the systematic review by Gazil et al., 2022 [51], two of their studies report the presence of red complex species in peri-implantitis lesions (*T. denticola* and *T. forsythia*) and five articles (*P. gingivalis*), as well as orange complex species (*P. intermedia*, *Bacteroides*, and *Filifactor* spp.). However, other authors, also included in the review, state that *F. nucleatum*, *P. intermedia*, and *P. gingivalis* are microorganisms that can also be found on healthy implants, although in lower relative abundance.

Bacterial diversity analysis showed that most of the PI samples from the nine donors in this study had higher Shannon index values than their concurrent PD sites. Only in patients 6 and 7 were the Shannon index values higher in PD samples. None of the clinical characteristics of these two patients correlated with this differential result. Interestingly, patients 5 and 7, who were administered antibiotics during the week prior to sampling, showed the greatest difference in Shannon index values between their respective PI and PD samples, which may indicate a differential effect of antimicrobial treatment in both pathologies. “The peri-implant microbiota is resistant to periodontal antimicrobial treatments”, a statement supported by the fact that the microbiota of peri-implantitis presents species such as *Veillonella* spp. and *Neisseria* spp. producing beta-lactamases, as well as other Gram-positive species identified in human peri-implantitis, such as *Streptococcus mitis* or *Streptococcus oralis*, that present penicillin-binding proteins conferring antibiotic resistance [53]. Nevertheless, the PCA could not identify a differential behaviour of samples from patients receiving antibiotic treatment (Figure 3).

The number of identified OTUs displayed the opposite trend to that of the Shannon index values. Most patients showed a higher number of OTUs in PD samples (103–147) compared to PI sites (63–111), although the highest number of OTUs was obtained in PI samples from patients 4 (150 OTUs) and 9 (202 OTUs), with no clear correlation with any of the clinical characteristics.

Recent studies based on the transcriptomic analysis of peri-implantitis and periodontitis sites from the same subject claim that the microbial compositions of both groups differed, with the microbiota of peri-implantitis being more complex than that of periodontal disease [15,16]. In addition, another study using the same analysis showed that plasmin receptor/glyceraldehyde-3-phosphate dehydrogenase gene activity was higher in peri-implant disease, suggesting significant changes in the pathogenic activity of the disease and the complexity of its microbiota [11].

Thanks to the development of new microbiological techniques, the knowledge of how these play a role in the pathogenesis of periodontal and peri-implant diseases is changing. However, it should not be forgotten that these diseases are determined by the interaction of the host and the environment, rather than by a specific group of bacteria. In fact, taking the literature as a reference, there is still no consensus on the pathogens associated with PI. In the present study, the periodontopathogens *Porphyromonas*, *Tannerella*, *Prevotella*, and *Treponema* were clearly more abundant in the PI samples than in the concurrent PD samples of these nine patients. These results align with the recent literature describing a higher diversity at PI sites and a higher relative abundance of *Prevotella* and *Treponema* species [52]. Similarly, a higher presence of important oral pathogens, including *P. gingivalis*, *P. intermedia*, and *T. forsythia*, has already been reported to be significantly higher in infected implants compared to healthy implants [18] or teeth [13] in studies using gene cloning and DNA–DNA hybridisation techniques. However, *Aggregatibacter*, generally associated with aggressive PD, was less abundant in PI sites, and could not be identified in four PI samples obtained from patients in whom this pathogen was present in concurrent PD lesions.

Our results indicate that the microbial communities of PI and PD obtained from the same patient present overlapping microbial profiles, while showing different proportions of important periodontopathogens. This is relevant both for daily clinical practice and for the direction of future research in the field of periodontal disease and peri-implantitis.

In the context of clinical practice, understanding variations in the microbiota between peri-implantitis and periodontal disease sites could have far-reaching therapeutic implications. Specifically, our findings may support the development of therapies targeting the predominant microorganisms in each condition, which could optimise treatment efficacy and improve the long-term management of these oral diseases. In addition, the identification of biomarkers associated with the characteristic microbiota of each disease could facilitate early detection, accurate diagnosis, and more accurate prognosis, allowing earlier and more targeted interventions to prevent the progression of periodontal disease and peri-implantitis.

In terms of future research, our findings provide a solid basis for further exploration of the underlying mechanisms that could contribute to the observed differences in microbiota between sites of peri-implantitis and periodontal disease. It is strongly recommended that future studies use multivariate analyses to further assess the impact of factors such as oral hygiene, diet, and genetics on the oral microbiota, which could enrich our understanding and allow for more effective personalisation of treatments and preventive strategies. Furthermore, longitudinal research is needed to investigate the temporal dynamics of the microbiota and its relationship to the progression of periodontal disease and peri-implantitis.

### Limitations of the Study

Despite the results obtained, it is critical to recognise the inherent limitations of our research design. Firstly, the sample size could be considered small, which may raise questions about the generalisability of our findings to larger populations. However, this restriction does not invalidate the robustness of our findings, but rather underlines the importance of future research with larger samples to confirm and extend our results. Furthermore, the absence of a control group without periodontal disease and peri-implantitis poses challenges in the interpretation of our observations, although our conclusions are supported by a thorough review of the existing literature. In addition, the use of a single sampling technique could introduce potential biases in the identification and characterisation of the oral microbiota. Importantly, despite these limitations, the use of NGS to investigate the 16S rRNA gene in our methodology allowed us to explore microbial diversity in unprecedented depth.

This not only strengthens the internal validity of our results, but also suggests the robustness of our inferences regarding the microbiota associated with periodontal disease and peri-implantitis. However, it is crucial to recognise that our conclusions are based on the clinical and demographic characteristics of our participants, which are representative of the general population. Furthermore, our findings are consistent with previous studies using larger samples, which further supports the robustness of our results. However, for a more complete understanding of the underlying mechanisms that may contribute to the observed differences in microbiota between sites of peri-implantitis and periodontal disease, future research is strongly recommended. These should include multivariate analyses to further assess the impact of factors such as oral hygiene, diet, and genetics on the oral microbiota. These additional considerations would enrich our understanding of the topic and strengthen the validity and clinical relevance of our results.

## 5. Conclusions

The data presented in this work indicate that PI and PD microbial communities obtained from the same patient are considerably different. Despite the alpha diversity indexes, such as the Shannon index, and the number of genera not indicating a clear correlation with the type of sample, the PI samples presented not only the highest prevalence of members of genera, which include oral periodontopathogens such as *Porphyromonas* and *Treponema*, but also the greatest bacterial diversity. However, a greater number of patients will be necessary to increase the depth of knowledge about these oral diseases triggered by bacteria and to be able to validate the results obtained in the first instance.

This work contributes to the current discourse on the aetiology and treatment of peri-implant infections, emphasising the importance of taking into account interpersonal variations in the oral microbiota when interpreting community profile studies.

Despite the progress provided by new microbiological techniques, it is important to keep in mind that periodontal and peri-implant diseases are the result of a complex interaction between the host and the environment, and not just a specific group of bacteria. Therefore, a deeper understanding of the underlying mechanisms contributing to these diseases is required to develop more effective and personalised therapeutic approaches.

## Figures and Tables

**Table 1:** Clinical information of the patients recruited in this pilot study.

Sample No.	Sex	Age	Smoker	Sprinkler	Antibiotic Uptake	CHX	Bleeding on Probing	Implant Connection	Implant Time	PI Time
1	Female	47	Yes	Yes	No	No	No	External	10 years	1 year
2	Male	67	Yes	Yes	No	No	No	External	6 years	2 years
3	Female	54	No (4 years)	Yes	No	No	Yes	External	8 years	3 years
4	Female	71	No	Yes	No	No	No	Internal	8 years	2 years
5	Female	59	Yes	Yes	Yes	Yes	No	Internal	12 years	1 month
6	Female	54	Yes	Yes	No	No	Yes	Internal	6 years	2 years
7	Male	45	No (6 years)	No	Yes	No	Yes	Internal	1 year	1 month
8	Female	47	N/A	Yes	No	Yes	Yes	External	5 years	6 months
9	Male	65	No	Yes	No	No	No	Internal	4 years	2 months

## Data Availability

The original contributions presented in the study are included in the article, further inquiries can be directed to the corresponding author.
